# Drug-Induced Liver Injury Caused by “Khat,” an Herbal Stimulant

**DOI:** 10.14309/crj.0000000000000480

**Published:** 2020-11-23

**Authors:** Pedro Palacios Argueta, Bashar Attar, Cameron Sikavi, Victoria Alagiozian-Angelova, Satya Mishra

**Affiliations:** 1 Internal Medicine Department, Cook County Health, Chicago, IL; 2 Department of Gastroenterology and Hepatology, Cook County Health, Chicago, IL; 3 Department of Pathology, Cook County Health, Chicago, IL

## Abstract

We describe a case of *Catha edulis* (Khat) drug-induced liver injury in a 28-year-old man from Yemen. The patient presented with jaundice, fatigue, and anorexia. Extensive workup, including liver biopsy, was performed. This is the first reported case in the United States without definite autoimmune hepatitis. Diagnosis requires high clinical suspicion and extensive workup. Increasing migration and differences in cultural practices lead to the need for an increased awareness of this type of cases, which is underreported.

## INTRODUCTION

Recreational chewing *Catha edulis* (Khat) is part of many cultures found in Eastern Africa, Somalia, and Yemen.^1^ Its global prevalence is unknown, and the calculated figure of 20 million users is likely underestimated.^2^ This plant is chewed in sessions lasting several hours, and its juice is then swallowed, causing a stimulant effect that mimics amphetamines.^3^ Its effects last 3–4 hours, and its use can vary from occasional (festivities and cultural activities) to daily. In Yemen, Khat use has a high social profile.^4^ The absorption of Khat in humans is described as a 2-compartment model. The first site is in the buccal mucosa, and the second one is in the small intestine.^3^ When consumed chronically, it can cause hepatocellular injury that can develop to autoimmune hepatitis (AIH) and fibrosis.^5^ Migration has led to the consumption of Khat in other regions of the world with reports of drug-induced liver injury (DILI) and acute liver failure in Somali immigrants in the United Kingdom, Belgium, and Canada.^6–8^ Its use and importation are banned in North America, Egypt, and most of Europe.^4^

## CASE REPORT

A 28-year-old man from Yemen, who emigrated to the United States 6 months prior, presented to the emergency department for 2 weeks of jaundice associated with fatigue, pruritus, loss of appetite, and postprandial vomiting. Medical history was significant for hospitalization in Yemen 2 years before this admission for similar symptoms that subsequently resolved. However, he could not recall exactly what happened. He endorsed recreational use of Khat consistently until 1 week before this admission but denied use of alcohol, recreational drugs, over-the-counter medications, herbs, or supplements. He had no family history of liver disease. On physical examination, vital signs were stable. The examination was significant for icterus alone, without other stigmata of chronic liver disease (CLD). There was no hepatosplenomegaly or ascites. Initial laboratory investigations revealed aspartate transaminase (AST) 920 U/L, alanine aminotransferase (ALT) 2,148 U/L, total bilirubin 6.1 mg/dL with direct bilirubin of 3.2 mg/dL, gamma-glutamyl transferase 124 U/L, alkaline phosphatase 184 U/L, and lactate dehydrogenase 534 (Table 1). Complete blood count, serum electrolyte levels, and coagulation studies were normal. Viral hepatitis serology was negative for hepatitis A, B, C, and E. Infection with cytomegalovirus, Epstein-Barr virus, herpes simplex viruses, and HIV was excluded as well. Urine toxicology was negative, and the acetaminophen level was <10.0 mg/mL.

**Table 1.**
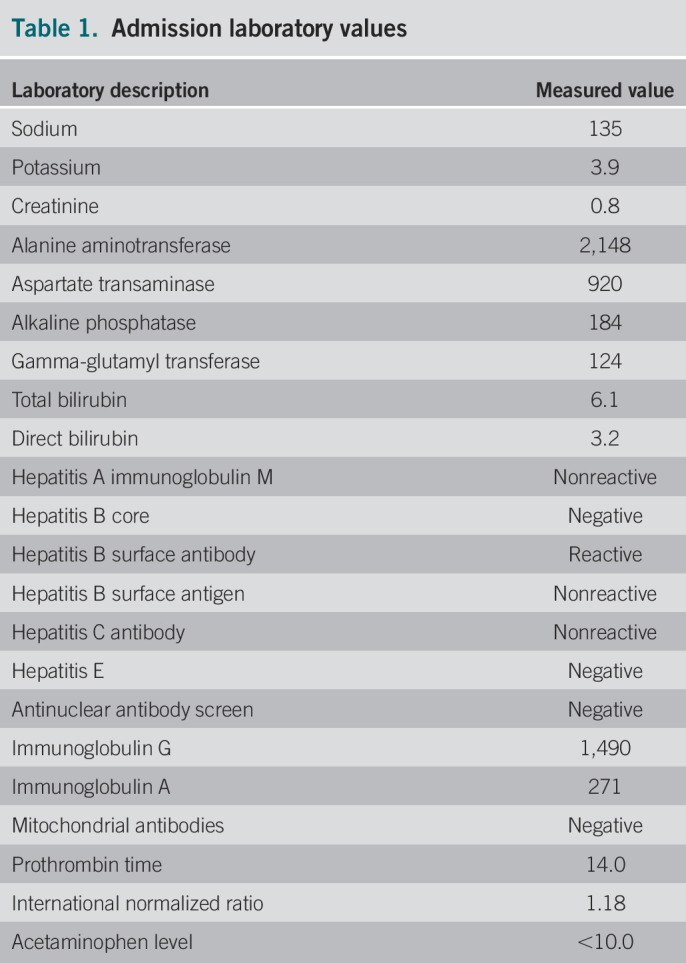
Admission laboratory values

Laboratory description	Measured value
Sodium	135
Potassium	3.9
Creatinine	0.8
Alanine aminotransferase	2,148
Aspartate transaminase	920
Alkaline phosphatase	184
Gamma-glutamyl transferase	124
Total bilirubin	6.1
Direct bilirubin	3.2
Hepatitis A immunoglobulin M	Nonreactive
Hepatitis B core	Negative
Hepatitis B surface antibody	Reactive
Hepatitis B surface antigen	Nonreactive
Hepatitis C antibody	Nonreactive
Hepatitis E	Negative
Antinuclear antibody screen	Negative
Immunoglobulin G	1,490
Immunoglobulin A	271
Mitochondrial antibodies	Negative
Prothrombin time	14.0
International normalized ratio	1.18
Acetaminophen level	<10.0

**Figure 1.**
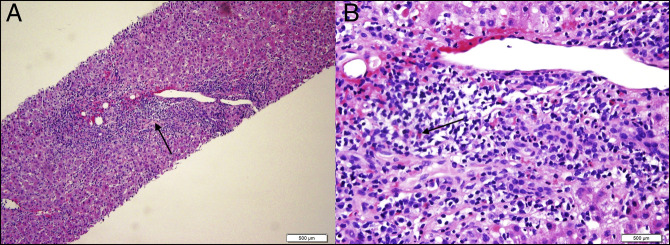
Liver biopsy showing (A) moderate portal and lobular inflammation with moderate plasma cells (black arrow) and minimal mixed macrovesicular and microvesicular stenosis (hematoxylin and eosin stain, 10× magnification) and (B) rare eosinophils (black arrow), lymphocytes, and plasma cells reaching the endothelium of the venule causing mild venulitis (hematoxylin and eosin stain, 40× magnification).

**Figure 2.**
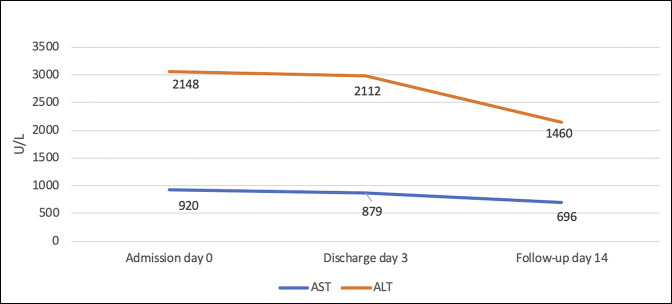
Liver transaminase levels. ALT, alanine aminotransferase; AST, aspartate transaminase.

Further workup did not show any evidence of hemochromatosis, Wilson disease, or alpha-1 antitrypsin deficiency. Autoimmune markers including antinuclear antibody, antimitochondrial antibody, anti–smooth muscle antibody, and anti-liver-kidney muscle antibody were negative. Liver ultrasound revealed coarse, echogenic liver echotexture, suggestive of diffuse liver parenchymal disease with a 1.5 × 1.5 cm homogenous hyperechoic lesion within the hepatic lobe. Further evaluation by a triple-phase abdominal computed tomography revealed liver hemangioma. The patient underwent liver biopsy that revealed moderate portal and lobular inflammation with moderate plasma cells and rare eosinophils, scattered apoptotic bodies, and minimal mixed macrovascular/microvascular steatosis (5%), consistent with drug-induced toxicity, viral hepatitis, and AIH (Figure 1). The patient was discharged home 3 days after admission with supportive care and with clear indications to abstain from Khat consumption. Liver function tests improved (AST 696 U/L and ALT 1,460 U/L from AST 920 and ALT 2,148 from admission) when repeated in the clinic 1 week after being discharged from the hospital (Figure 2). However, the patient was unavailable to follow-up.

## DISCUSSION

Cathinone, an alkaloid present in the plant, is absorbed during chewing and creates a state of alertness and euphoria similar to amphetamines.^1,3^ Its use can lead to acute hepatitis, but it also has been linked to CLD.^9^–^13^ Six cases of fulminant hepatic failure have been reported in the United Kingdom and 1 case in Belgium.^7,11^ One case has been reported so far in the United States, in a patient from Yemen where his presentation was consistent with DILI with positive markers for underlying AIH.^14^ It is reported in Ethiopia, a region of high Khat consumption, that more than 50% of decompensated CLD admissions are of unknown etiology.^15^ A subsequent study showed a dose-dependent positive correlation between this plant and the development of CLD with a 2-fold increased risk independent of hepatitis B, alcohol, and age.^13^

Resolution of liver injury after cessation and relapse after re-exposure have been observed.^10,11,16^ This is likely the case in our patient who was previously admitted in his country of origin where he consumed Khat regularly and resumed its use after his immigration to the United States, causing recurrence with severe acute hepatitis. The mechanism of liver injury is unknown, and several theories have been developed including: (i) an autoimmune process as low titers of autoantibodies and histological features of AIH^9,16,17^ have been reported; (ii) toxic injury related to Khat as Khat alkaloids are extensively metabolized in the liver by the enzyme cytochrome P450 2D6, and accumulation of these metabolites and individual susceptibility may play a role in liver injury and progression to CLD^3,18^; and (iii) toxic injury related to contaminants in Khat such as pesticides, heavy metals, and fungi.^19,20^

Although the serologic workup was negative in our patient, AIH could not be completely excluded based on his liver histology. The AIH score based on the simplified criteria for diagnoses of AIH was 5, suggesting possible AIH.^21^ The unique characteristic of our case is the lack of objective data consistent with AIH, including the liver biopsy that was not diagnostic for AIH. Similar to the case reported in Belgium where liver biopsy showed the absence of plasma cells and a high number of eosinophils, our case had a mixed pattern with moderate plasma cells and rare eosinophils.^7^ This is the first report of DILI secondary to Khat without features of autoimmune liver disease in the United States. The use of Khat is generally underreported because it is illegal in the United States, the United Kingdom, Canada, and Europe. Khat-induced liver injury should be suspected in patients from Eastern Africa and Arab origin or descent when no other explanation is present in the setting of acute liver injury.

## DISCLOSURE

Author contributions: All authors contributed equally to this manuscript. P. Palacios Argueta is the guarantor of the article.

Financial disclosure: None to report.

Informed consent was obtained for this case report.
